# Two Residues in the Basic Region of the Yeast Transcription Factor Yap8 Are Crucial for Its DNA-Binding Specificity

**DOI:** 10.1371/journal.pone.0083328

**Published:** 2013-12-16

**Authors:** Catarina Amaral, Catarina Pimentel, Rute G. Matos, Cecília M. Arraiano, Manolis Matzapetakis, Claudina Rodrigues-Pousada

**Affiliations:** 1 Genomics and Stress Laboratory, Instituto de Tecnologia Química e Biológica António Xavier, Universidade Nova de Lisboa, Oeiras, Portugal; 2 Control of Gene Expression Laboratory, Instituto de Tecnologia Química e Biológica António Xavier, Universidade Nova de Lisboa, Oeiras, Portugal; 3 Biomolecular NMR Laboratory, Instituto de Tecnologia Química e Biológica António Xavier, Universidade Nova de Lisboa, Oeiras, Portugal; University of South Florida, United States of America

## Abstract

In *Saccharomyces cerevisiae*, the transcription factor Yap8 is a key determinant in arsenic stress response. Contrary to Yap1, another basic region-leucine zipper (bZIP) yeast regulator, Yap8 has a very restricted DNA-binding specificity and only orchestrates the expression of *ACR2* and *ACR3* genes. In the DNA-binding basic region, Yap8 has three distinct amino acids residues, Leu26, Ser29 and Asn31, at sites of highly conserved positions in the other Yap family of transcriptional regulators and Pap1 of *Schizosaccharomyces pombe*. To evaluate whether these residues are relevant to Yap8 specificity, we first built a homology model of the complex Yap8bZIP-DNA based on Pap1-DNA crystal structure. Several Yap8 mutants were then generated in order to confirm the contribution of the residues predicted to interact with DNA. Using bioinformatics analysis together with *in vivo* and *in vitro* approaches, we have identified several conserved residues critical for Yap8-DNA binding. Moreover, our data suggest that Leu26 is required for Yap8 binding to DNA and that this residue together with Asn31, hinder Yap1 response element recognition by Yap8, thus narrowing its DNA-binding specificity. Furthermore our results point to a role of these two amino acids in the stability of the Yap8-DNA complex.

## Introduction

Baker’s yeast *Saccharomyces cerevisiae* contains a family of eight basic region-leucine zipper (bZIP) transcription factors involved in the response to stress, designated as the Yap family [1]. Yap1, the best studied member, is activated by thiol reactive chemicals and peroxides, which leads to its nuclear sequestration [2]. This transcription factor was identified due to its ability to bind a DNA sequence containing the simian virus 40 (SV40) sequence TGACTAA [3]. Later Yap1 was shown to bind more efficiently the sequence TTACTAA, that is here designated Yap response element (YRE) [4]. Recently, a high throughput analysis revealed that the YRE usually contains an adenine or cytosine at the 5’ end, (A/C)TTACTAA [5]. In addition, Yap1 is also able to bind the sequences TGACTAA, TTAGTCA and T(T/G)ACAAA [6] [7].

Another member of the Yap family is Yap8, the main regulator of the response to arsenic stress [8,9]. When yeast cells are exposed to arsenic compounds, Yap8 accumulates in the nucleus where it drives the expression of *ACR2* and *ACR3* encoding, respectively, an arsenate reductase and an arsenite efflux pump [10]. *ACR2* and *ACR3* genes share a common promoter, containing a 13 base pair sequence, TGATTAATAATCA, that was shown to be recognized by Yap8 [9,11] and which is hereafter designated as Yap8 response element (Y8RE). Both the core element (TTAATAA) and the flanking regions of the Y8RE are crucial for Yap8 binding and for *in vivo* activation of its targets [11]. 

Yap1 also plays an important role in arsenic detoxification. Indeed, Yap1 regulates the expression of *YCF1* gene, which encodes a vacuolar ATP binding cassette (ABC) transporter that functions as a vacuolar glutathione-S-conjugated pump [12–14]. Moreover, we have previously shown that this transcription factor is crucial to mitigate the oxidative damages caused by arsenic stress [13]. Interestingly, it was shown that Yap1, when overexpressed, stimulates the expression of *ACR3*, upon exposure to arsenite and that Yap1 may compete with Yap8 for binding to the *ACR3* promoter, but Yap1 is unable to act as a potent activator [15].

Although, Yap1 and Yap8 share a highly conserved basic region predicted to bind DNA, these transcription factors exhibit distinct ranges of specificities. Yap1 activates the transcription of many genes and appears to have a broad specificity over the recognized DNA sequences [4,6,7]. Conversely, Yap8 has a very narrow specificity and only orchestrates the expression of *ACR2* and *ACR3* genes [11]. 

In this work, *in vivo* and *in vitro* approaches together with bioinformatics analysis were used to study the relevance of conserved and non-conserved residues of the basic region of Yap8 for DNA binding. Our data indicate several conserved amino acid residues important for Yap8 binding to DNA. In addition we found that at least one non-conserved residue of Yap8, Leu26, is important for the Y8RE recognition. Furthermore, in an attempt to understand the mechanisms underlying Yap8 specificity, we have substituted the Yap8-specific residues of the DNA binding region of Yap8 by the corresponding amino acids of Yap1. Interestingly, our results suggest that in Yap8, Leu26 together with Asn31 hinder the YRE recognition, possibly explaining the limited specificity of Yap8 transcription factor.

## Materials and Methods

### Plasmids, strains and growth conditions

The *S.cerevisiae* strains used were FT4: *MATa ura3-52; trp1-∆63; his3-∆200; leu2::PET56* [16], FT4*yap8: MATa; ura3-52; trp1Δ63; his3-Δ200; leu2::PET56; yap8::KAN* [13] and strains BY4741: *MATa*; *his3∆1*; *leu2∆0*; *met15∆0*; *ura3∆0* (EUROSCARF), BY4741*yap8: MATa; his3Δ1; leuΔ0; met15Δ0; lys2Δ0; ura3Δ0; YPR199c::kanMX4* [8]. Yeast strains were grown at 30°C in YPD medium (1% yeast extract, 2% bacto-peptone 2% glucose and 1.5% of agar all from Difco). For plasmid selection synthetic dropout medium (SD) (0.67% yeast nitrogen base without amino acids, 2% glucose and 1.5% bacto-agar for solid media) supplemented with required amino acids and bases was used. 

*E. coli* was grown at 37°C in LB broth (1% NaCl, 0.5% yeast extract, 1% bacto-tryptone, all from Difco). Selection of recombinant clones was performed by growth in the presence of 50µg/mL kanamycin or 100μg/mL ampicillin (Sigma). 

All the plasmids used in this work are listed in Table S1 in Text S1.

Yap8 mutations were generated by site-directed mutagenesis as detailed in [8] with the primers presented in Table S2 in Text S1.

### Phenotypic analysis

Yeast strains were grown in SD medium supplemented with Na_2_HAsO_4_ .7H_2_O (As(V)) or NaAsO_2_ (As(III)), (Sigma). Exponential phase cells were diluted in phosphate buffered saline (PBS) in order to spot 2500, 250 and 25 cells. Growth at 30°C was recorded after three days. For *in vivo* DNA binding assay the wild type strain FT4 was transformed with a plasmid containing the YRE or the Y8RE inserted as the upstream activating sequence of *HIS3*, and with a plasmid overexpressing Yap1, Yap8 or Yap8 mutated versions. Transformation with the empty vector was used as a control. Cells were spotted as described above in plates with SD medium with or without histidine.

### Cell extracts and protein purification

BL21(DE3) *E. coli*, expressing the selected mutants were lysed using a French press at 900psi (1psi=6.9kPa) in buffer B (10mM phosphate buffer pH 7.5, 100mM NaCl) with 0.2mM PMSF. The extracts were clarified by centrifugation at 15kg for 20min and the supernatant was frozen at -20°C after addition of glycerol to a final concentration of 50% (v/v). Concentrations of total protein were determined by DC (BIORAD) in a microplate. Quantification was verified by running 0.5µg of each extract in SDS-PAGE (15% tricine gel) and visualized by Coomassie Blue staining. 

Purification of the Yap8bZIP and respective mutants, Yap8bZIPL26A and Yap8bZIPL26N-N31R (Yap8bZIPLNNR) was performed by histidine-affinity chromatography using HisTrap HP Columns (GE Healthcare) and ÄKTA FLPC system (GE Healthcare). Cell suspensions were lysed using a French press at 900psi (1 psi=6.9 kPa) in the presence of 0.1mM PMSF. The crude extracts were clarified by a 30min centrifugation at 18000*g*. The clarified extracts were then added to a 1ml HisTrap column equilibrated in buffer A (20mM phosphate buffer, pH7.5 and 0.5M NaCl) plus 10mM imidazole. Protein elution was achieved by a continuous imidazole gradient (from 10 to 500mM) in buffer A. The fractions containing the purified protein were buffer-exchanged to buffer B (10mM phosphate buffer, pH 7.5, 100mM NaCl) using a PD10 desalting column (GE Healthcare). Eluted proteins were concentrated by centrifugation at 3000*g* for 2h at 4°C with Vivaspin15R of 2000Da molecular-mass cut-off. Protein concentrations were determined by spectrophotometry in a Nanodrop ND-2000C (Thermo Scientific) and 50% (v/v) glycerol was added to the final fractions before storage at −20°C. Concentration and purity were verified by running a 0.5μg sample of each purified protein in SDS-PAGE (15% tricine gel) and was visualized by Coomassie Blue staining.

### Electrophoretic mobility shift assay

The purified protein was incubated with the DNA in binding buffer (5mM Hepes, pH 7.5, 0.05Mm EDTA, 0.014% Nonidet P40, 5% glycerol) for 30min, at 23°C. The mixture was loaded in a 10% non-denaturing gel of polyacrylamide and run in TBE 0.5x at 100V. The gel was stained with TBE 0.5x containing ethidium bromide. 

### Surface Plasmon Resonance analysis of the protein-DNA complexes dissociation constants

Surface Plasmon Resonance experiments were performed using the Biacore 2000 system (GE Healthcare). 5’ biotinylated DNA oligomers, carrying the Y8RE, were immobilized in one of the cells of a streptavidin coated sensor chip SA (GE Healthcare). The chip was previously prepared, according to the manufacturer’s instructions. Another flow cell was left without immobilized DNA and used as a control. All the assays were run at 25°C in running buffer (5mM Hepes pH7.5, 5mM of phosphate buffer pH 7.5, 0.05mM EDTA, 50mM NaCl, 0.014% Nonidet P40 and 5% glycerol). Different concentrations (1000, 500, 375, 250 and 200nM) of the purified bZIP protein (Yap8bZIP, Yap8bZIPL26A or Yap8bZIPLNNR) were injected into the chip cells for 5 minutes, with a flow rate of 20µl/min. Dissociation was monitored for 15 minutes in running buffer. Before each new protein/concentration injection, proteins bound to the oligomers were removed by washing for 120s (1M NaCl, 50mM NaOH). After each cycle of association/dissociation/washing, the signal was stabilized for 5 min prior to new protein injection. 

All experiments were made in triplicate. Rate constants were calculated using the BIAEVALUATION 3.0 software package by fitting the sensograms obtained for each protein to a 1:1 Langmuir binding. 

### Real time-PCR

BY4741 *yap8* were collected at early log-phase and either untreated or exposed to arsenate (Na_2_HAsO_4_.7H_2_O) during the time and at the concentrations indicated. RNA was extracted using phenol-chloroform, treated with DNaseTurbo (Ambion), and purified (Quiagen, RNAeasy). cDNA was synthesized from 1µg of RNA, using Transcriptor Reverse Transcriptase. qRT-PCR reactions were performed using LightCycler 480 Green I Master in the LightCycler480 (all from Roche). Relative standard curves were constructed for each gene, using serial dilutions of cDNA. The relative expression of the genes was calculated by the relative quantification method with efficiency correction, using the LightCycler Software 4.1. Actin 1 (*ACT1*) was used as a reference gene. All assays were made at least in biological triplicates. The oligomers used in this assay are listed in Table S2 in Text S1.

### Yap8bZIP - DNA interaction model

The structural model of Yap8bZIP was derived using comparative modeling methods, with the program MODELLER release 6 [17]. For this, the X-ray structure of Pap1 from *S. pombe* was used. The PDB was searched using the amino acid sequence of Yap8 and only the PDB for Pap1 was found (for the basic and leucine zipper domains) (1gd2)[18]. This structure was chosen as template, which sequence shows 44% sequence identity to Yap8 sequence. Then, the alignment was changed in an iterative process, and new structural models were derived until its quality, assessed using the program PROCHECK [19] was found to be satisfactory. 

For the DNA model, bases of the Pap1 site were replaced by the corresponding bases of the Y8RE, with an extra base in the center (TGATTACATAATCA), using 3DNA [20]. The central cytosine was kept, in order to preserve the distance between the protein chain and the DNA. 

## Results

### The basic region of Yap8 contains several conserved and Yap8-specific amino acid residues that may interact with DNA

**Figure 1:**
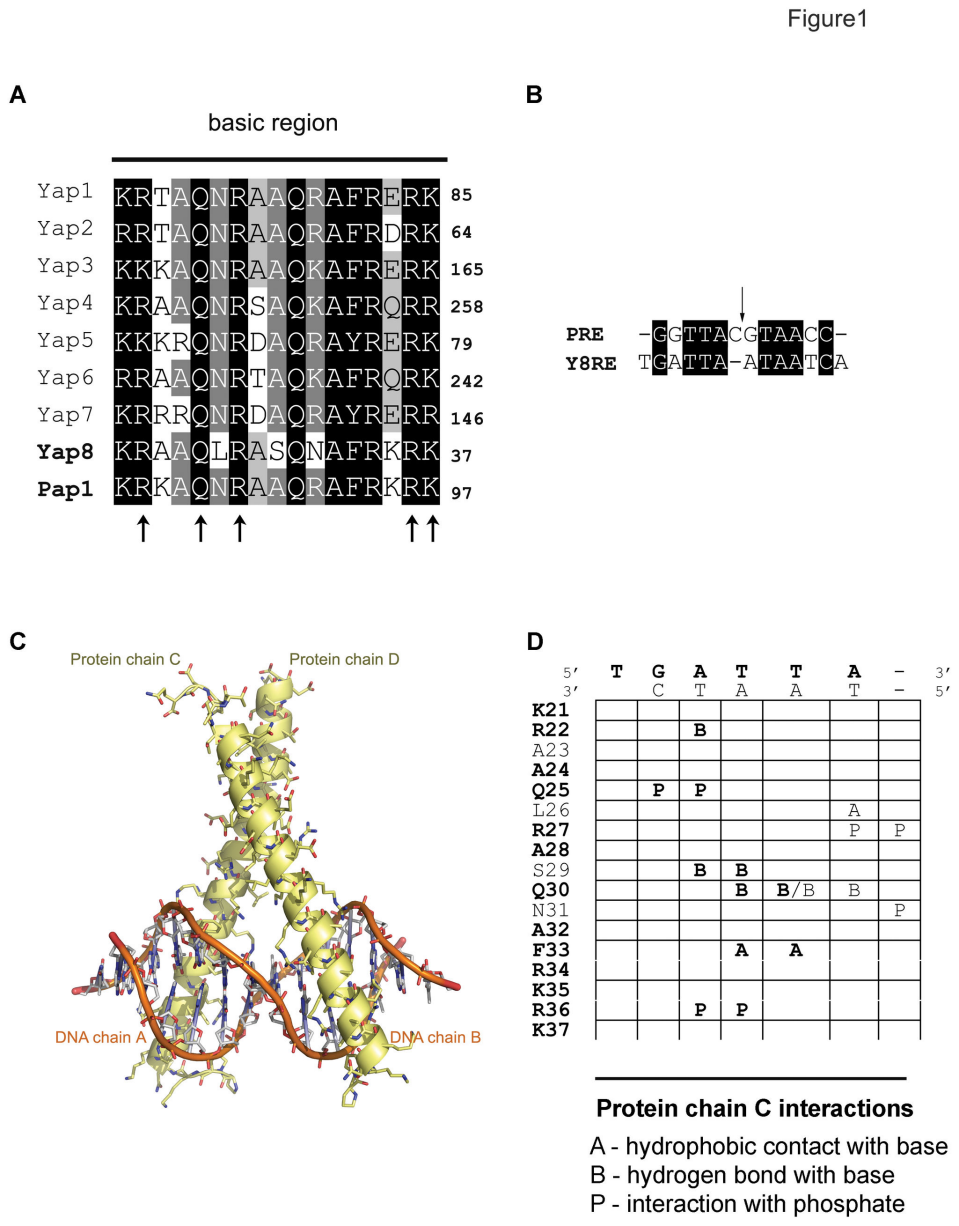
Structural features of the basic region of Yap8. (**A**) Sequence alignment of the basic region of the eight Yap family members of *S. cerevisiae* with their orthologue from *S. pombe*: Pap1. The four levels of shade correspond to 100% of similarity (black); 80-90% of similarity (grey with white symbols); 60-79% of similarity (grey with black symbols) and less than 60% of similarity (not shaded). Arrows indicate the conserved amino acids of Yap8 that have been herein studied. Alignment with Clustal (whttp://www.ebi.ac.uk/Tools/msa/clustalw2) and edition with Genedoc (http://www.psc.edu/biomed/genedoc). (**B**) Sequence alignment of the DNA sequence recognized by the basic region of Pap1 with the corresponding region of Yap8. Similar bases are colored in black. The palindrome dyad axis of Pap1 site is indicated with an arrow. (**C**) Representation of the Yap8 and DNA models showing the possible positioning and identification of the different chains. Image created using PyMOL (The PyMOL Molecular Graphics System, 2006 DeLano Scientific LLC). (**D**) Graphical representation of the possible interactions between Yap8 and the DNA as predicted by the protein homology and DNA model. The bZIP residues conserved between Yap8 and Pap1 are in bold. Protein contacts with the DNA strand B are in bold whereas contacts with the DNA strand A are in grey. PRE- refers to the Pap1 response element and Y8RE- refers to the Yap8 response element.

The bZIP transcription factors contain a region rich in basic amino acids that is relevant to the specificity and affinity of these regulators to DNA [4,18,21]. As a first approach to understand the specificity and affinity of Yap8 to the Y8RE, we aligned the basic region of the Yap family with the corresponding region of the Yap1 functional orthologue from *Schizosaccharomyces pombe*, Pap1. As depicted in Figure 1A, Yap8 shares a similar basic region with the other Yap members and Pap1. Notably, Yap8 basic region sequence contains three distinct amino acid residues (Leu26, Ser29, and Asn31) at sites of highly conserved positions in the other Yap family members and Pap1. The corresponding amino acids in Pap1 recognize bases and/or interact with phosphates [18]. Hereafter we will designate them as the Yap8-specific residues. 

In order to evaluate the possible contribution of each amino acid of the Yap8 basic region to the interaction with DNA, a protein-DNA model needed to be generated. For this we chose to use the known crystal structure of Pap1-DNA (PDB code 1GD2)[18] as a template due to the high similarity of the respective basic regions of Yap8 and Pap1 as well as the DNA fragments they recognize (Figure 1A and B). Our model assumes that Yap8 functions as a homodimer based on previous observations that Yap8 can interact with itself [22]. 

To build the model of the bZIP domain of Yap8 we used homology modeling, based on the alignment with Pap1 (Table S3 in Text S1), with the program MODELLER [17]. The resulting model quality was evaluated by PROCHECK [19] and visual inspection.

 The Yap8 DNA fragment was built on the Pap1 DNA backbone by substitution of the Pap1 response element (PRE) bases to those of Y8RE using the program 3DNA [20]. To maintain the alignment of the two DNA sequences and preserve the distances between DNA and protein monomers, we had however to convert the pseudo palindrome Y8RE to the PRE palindrome recognized by Pap1 (Figure 1B). A cytosine was introduced in the middle of the Yap8 DNA sequence resulting in the TGATTACATAATCA site instead of the native TGATTAATAATCA. This is justified by the observation that proteins that are able to bind both palindromic and pseudo palindromic sequences, induce a distortion on the DNA structure of the palindrome [21,23]. However, the protein-DNA contacts in both cases remain the same. The resulting model of the complex was found to be practically symmetric (Figure 1C). Regarding Yap8 protein chain D, we observed however, a close proximity between one side chain amino acid residue and the DNA. As such, only the Yap8 protein chain C of the model was considered to evaluate the contacts with DNA.

In addition, we did not attempt to construct a hydrated model of the protein-DNA complex, although nearly all protein-DNA complexes whose structures have been determined include multiple “trapped” waters. These water molecules can, and often do, extend the interactions of the residues beyond direct base contacts. Because of the difficulty of modeling water mediated protein-DNA interactions we opted to simply assess general proximity between Yap8 amino acid side chains and the DNA bases, practically reducing the effective resolution of the model. 

We then proceeded to obtain experimental results to support the proposed functional role of the conserved and Yap8-specific amino acids.

### Relevance of the basic region conserved Yap8 amino acid residues

**Figure 2:**
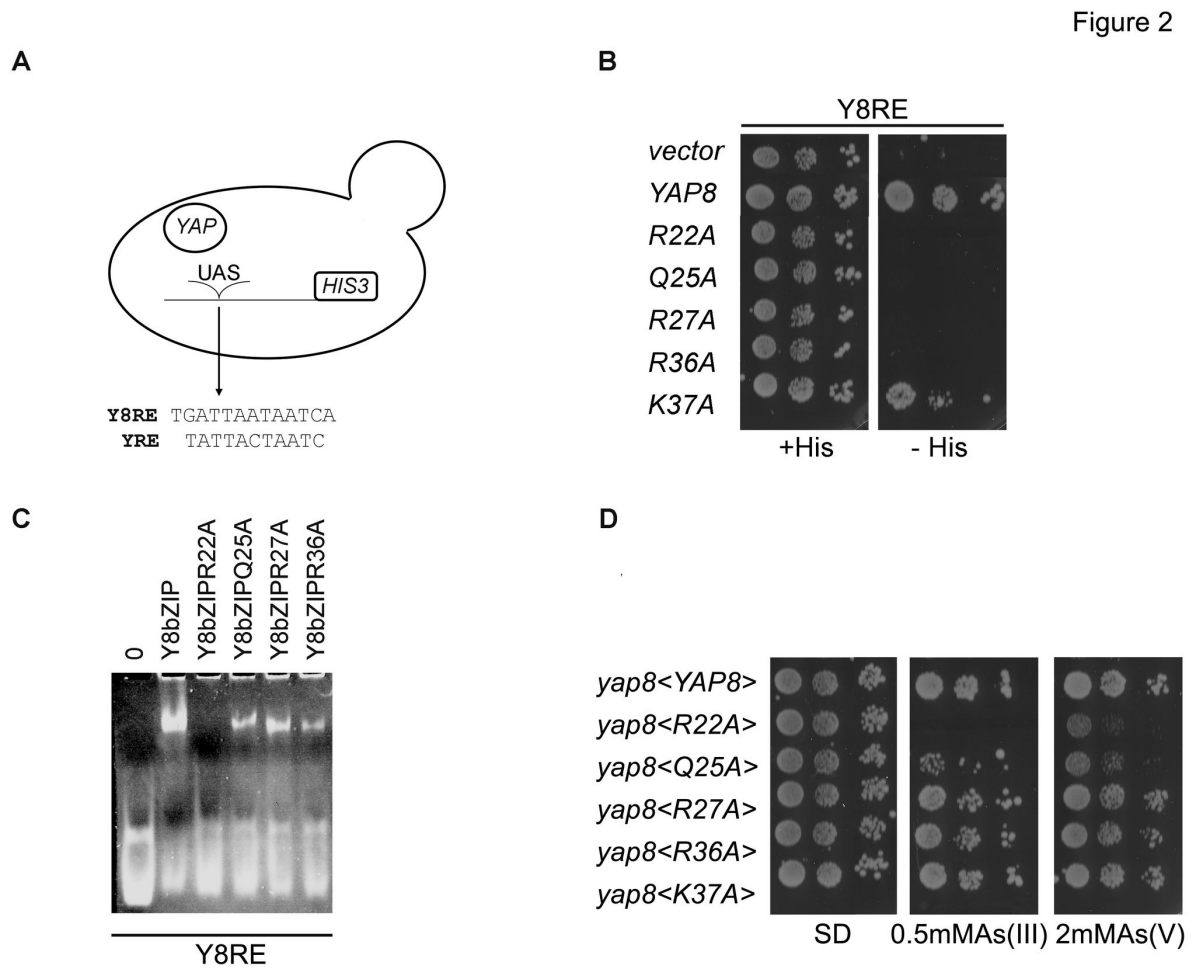
Several conserved amino acid residues of Yap8 are relevant for its DNA binding and activity. (**A**) Schematic representation of the assay used to monitor the *in* *vivo* binding of Yap8 to its cognate DNA. Plasmids encoding either Yap8 or Yap8 mutated versions were overexpressed in a wild-type strain containing the HIS3 gene under the control of Yap or Yap8 response element (YRE and Y8RE respectively). (**B**) Replacement of Arg22, Gln25, Arg27 and Arg36 by Ala, impair Y8RE recognition. Strains were spotted on minimal media with or without histidine (+His, -His, respectively). (**C**) Mutation of Arg22 to Ala prevents Yap8 binding to Y8RE. Purified Yap8bZIP and Yap8 mutated proteins (Yap8bZIPR22A, Yap8bZIPQ25A, Yap8bZIPR27A and Yap8bZIPR36A) were incubated with the DNA Y8RE and analyzed by electrophoretic mobility shift assay (EMSA), as described in materials and methods. (**D**) The conserved amino acid residue mutants have different capacities to rescue a *yap8* strain under arsenic stress. Wild-type (B) or *yap8* (D) strains were transformed with the empty plasmid YEplac112 (<vector>) or with plasmids overexpressing the native YAP8 or the indicated YAP8 mutated versions. Serially diluted cells were spotted onto selective dropout (SD) media, as indicated.

In order to validate the Yap8bZIP-DNA model information we mutated to Ala some of the conserved or partially conserved residues of the Yap8 basic region predicted to interact with DNA, namely Arg22, Gln25, Arg27 and Arg36, (Figure 1D). The DNA binding capacity of the mutants was next assayed by means of an indirect approach, as described in [4]. A strain expressing the *HIS3* gene under the control of the Y8RE was transformed with a multi-copy plasmid encoding the four Yap8 mutants referred above (Figure 2A). Recognition of the DNA sequence by overexpressing *YAP8* gene or its mutated versions allowed the strain to grow in medium without His. However, all the tested *YAP8* mutated versions (Arg22A, Gln25A, Arg27A and Arg36A) are not able to rescue the growth phenotype observed in the absence of His (Figure 2B). We have as well determined the transactivation potential (Figure S3) that remains almost identical to the one of the wild-type. As predicted by the model, Figure 1D indicates that the mutation of these amino acids may disrupt the binding to DNA. 

To further confirm these results, we next used an assay to directly monitor whether Yap8 mutations affect DNA binding. As such, we expressed in *E. coli* the predicted bZIP region of Yap8, Yap8bZIP. The protein was purified and analyzed by circular dichroism (CD) spectroscopy, in order to verify its correct folding (Figure S5). The CD spectra indicated that the protein is, as expected, mainly α*-*helical and, as previously observed for another bZIP transcription factor, Gcn4, it becomes more α-helical at lower temperature [24]. Yap8 conserved residues (Arg22, Gln25, Arg27 and Arg36) were then mutated to Ala in order to analyze the effect of these mutations in the Yap8 DNA binding capacity. Using electrophoretic mobility shift assay (EMSA), we observed that the mutation R22A completely abolishes Yap8 binding to Y8RE (Figure 2C). The Yap8bZIPQ25A, Yap8bZIPR27A and Yap8bZIPR36A mutants, however, are able to bind DNA, but probably with lower efficiency as suggested by the fainter band observed (Figure 2C). Accordingly, in experiments where *YAP8* and *YAP8* mutated versions were reintroduced in a *yap8* mutant strain, overexpression of *YAP8R22A*, did not endow *yap8* strain with the capacity to grow in the presence of arsenite and arsenate (Figure 2D). Under arsenite concentrations of 0.5mM, the mutant Yap8bZIPQ25A partially alleviates the growth phenotype of a *yap8* strain. In the presence of arsenate or higher arsenite concentration, however, this mutant is no longer able to rescue the phenotype (Figure 2D). The remaining mutated Yap8 versions (Yap8R27A, Yap8R36A) appear to fully restore Yap8 activity (Figure 2D).

Similar to Pap1, the Yap8 residues Gln25, Arg27 and Arg36 are predicted to interact with DNA phosphates (Figure 1D). In Pap1 the residue corresponding to Arg22 interacts directly as well as *via* a water molecule with several bases. In the Yap8bZIP-DNA model, this amino acid is also predicted to interact with the DNA bases (Figure 1D). In further agreement with our model, the mutation to Ala of one conserved residue of the basic region that was predicted not to interact with DNA (Lys37, Figure 1D) neither affects the binding of Yap8 to DNA as shown in *vivo* and *in vitro* (Figure 2B and S4), nor the Yap8 function (Figure 2D). We showed that there are no differences in the relative abundance of the Yap8 derivative proteins, as evaluated by Western blot (Figure S1).

Altogether our results experimentally support the Yap8bZIP-DNA homology model predictions (Figure 1D) and suggest that the conserved residues are probably functionally conserved, across different bZIP Yap family members.

### Significance of the basic region Yap8-specific amino acid residues

**Figure 3:**
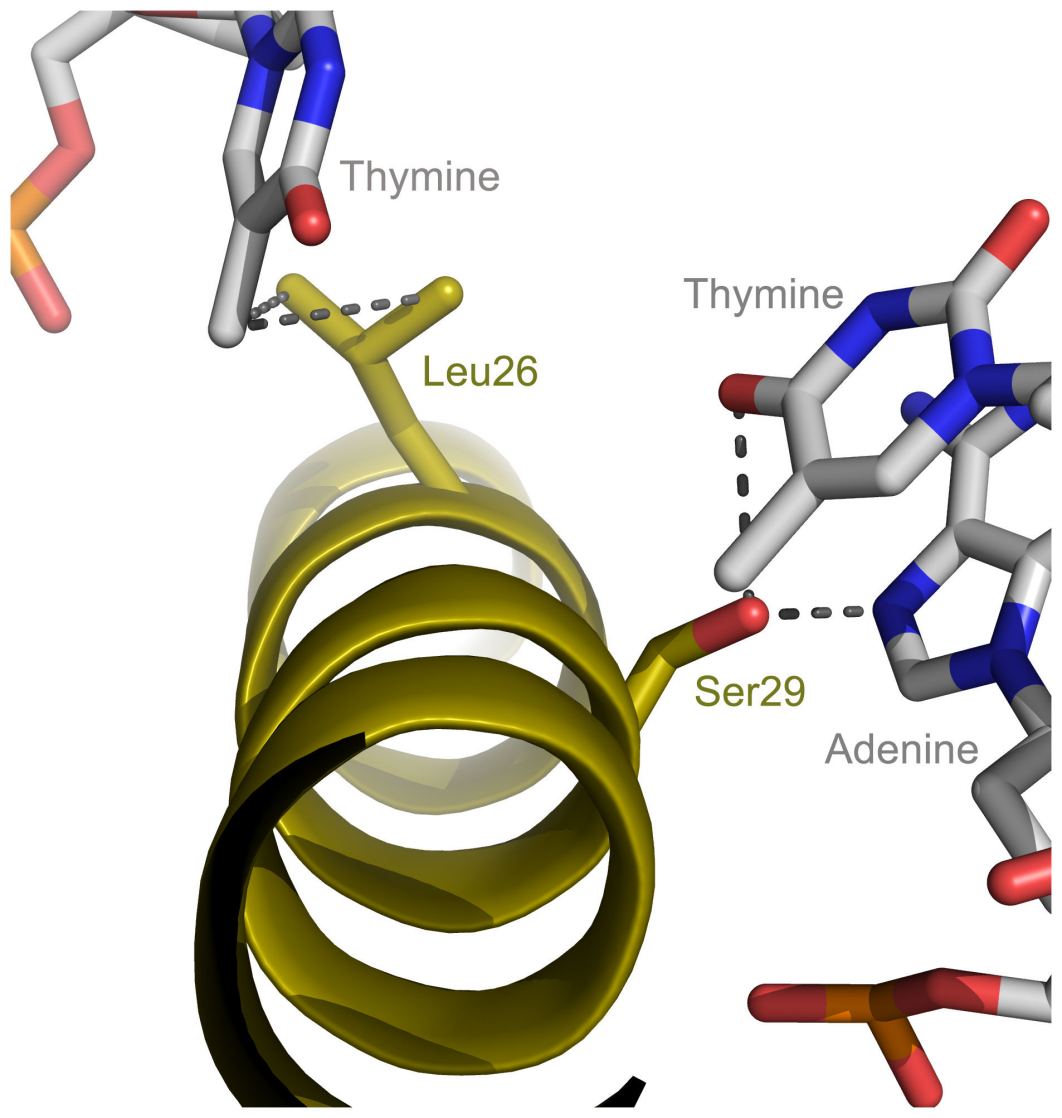
Model for Leu26 and Ser29 proximity to bases. PyMOL (The PyMOL Molecular Graphics System, 2006 DeLano Scientific LLC) was used to assess the possible interactions between Yap8 amino acid residues side chains and the Y8RE. Leu26 may be in the vicinity of the fifth thymine of the Y8RE (TGATTATTAATCA), whereas Ser29 may be close to the first adenine and second thymine of the Yap8 response element (Y8RE) (TGATTAATAATCA).

 According to our model, two out of the three Yap8-specific amino acid residues -Leu26 and Ser29 - may interact with the DNA bases (Figure 1D). Leu26 is in the vicinity of the methyl group of the last thymine that base pairs with the second adenine of the Y8RE (Figure 3). In that position, Pap1 and the other Yap members contain a very conserved Asn which, according to the Pap1 b-ZIP crystal structure interacts directly with the adenine that forms a base pair with the first thymine of the Pap1 site [18]. The second Yap8-specific residue, Ser29, is in the proximity of the first adenine of the Y8RE (Figure 1D and 3). In Pap1 and other Yap members Ser29 of Yap8 is replaced by an Ala (Figure 1A), which is known to form a hydrophobic contact with the methyl group of the first thymine of the Pap1 site [18].

**Figure 4:**
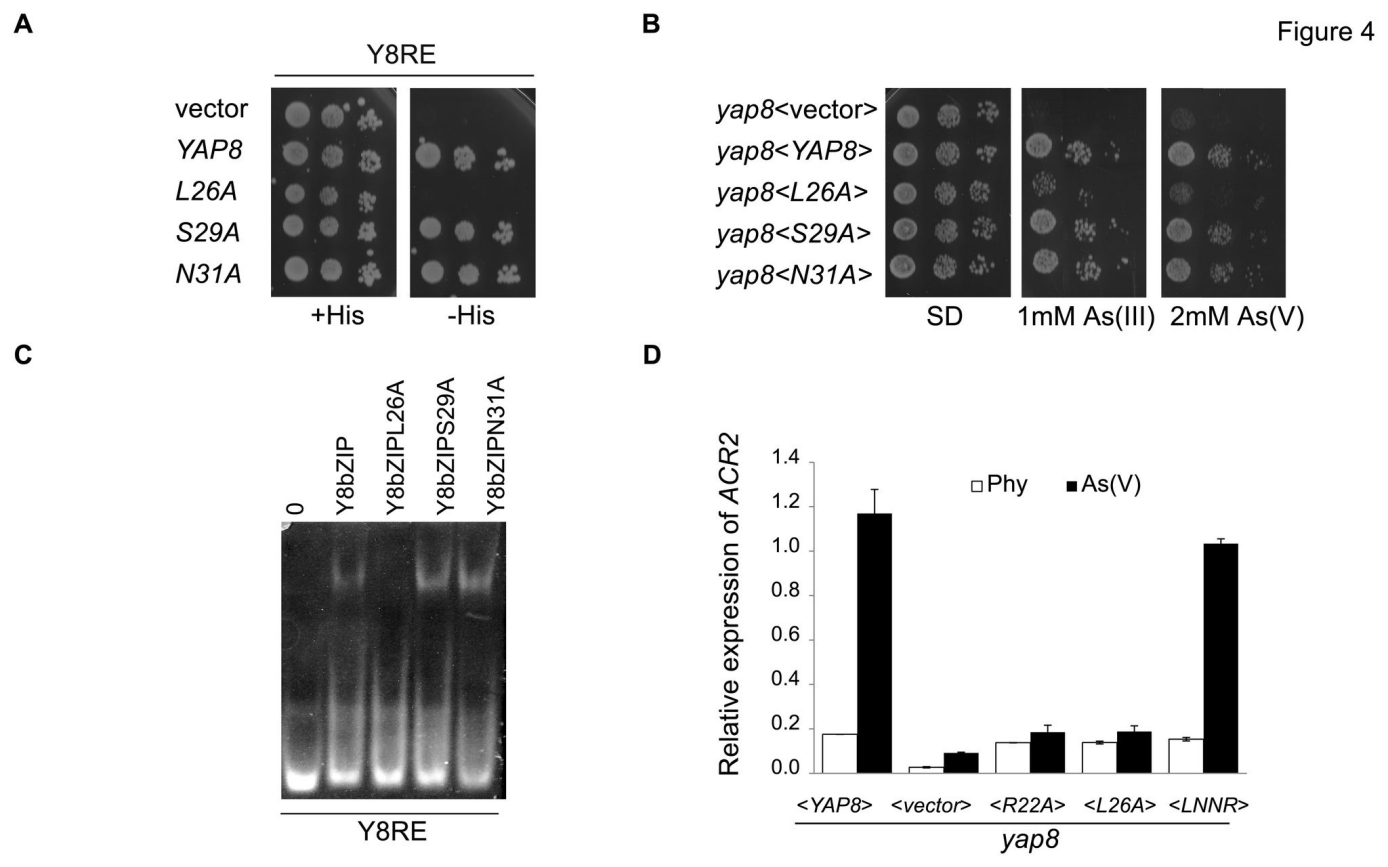
The Yap8-specific Leu26 residue is important for its activity. (**A**) Replacement of the Yap8-specific amino acid Leu26 by Ala eliminates binding *in* *vivo* to the Yap8 response element (Y8RE). Strains overexpressing native or Yap8 mutated versions were spotted on selective dropout with or without histidine (+His, -His). (**B**) Leu26 is the only Yap8-specific amino acid essential for Yap8 activity. Wild-type (A) or *yap8* (B) strains were transformed with the empty plasmid YEplac112 (<vector>) or with plasmids overexpressing the native YAP8 or the indicated YAP8 mutants. Serially diluted cells were spotted onto selective dropout (SD), as indicated. (**C**) Replacement of the Yap8-specific amino acid Leu26 by Ala eliminates binding *in* *vitro* to the Yap8 response element (Y8RE). Total extracts of *E.coli* harboring the vector encoding the indicated protein (Y8bZIP or mutated versions) were obtained and treated with RNase. Equal amounts of total protein were incubated with the same amount of the DNA Y8RE and were analyzed by electrophoretic mobility shift assay (EMSA) as described in materials and methods. (**D**) The *yap8* mutant strain with the plasmid encoding Yap8L26A is not capable of inducing transcription of ACR2 as is the case with the strain harboring the plasmid encoding the wild-type Yap8, upon arsenic stress. The *yap8* cells transformed with the empty pRS416 (<vector>) or with plasmids encoding either the native Yap8-c-myc or the indicated Yap8-c-myc mutated versions, were grown until exponential phase and up-shifted to 1mM arsenic for 1h. The expression of the ACR2 gene was assessed by quantitative RT-PCR.

Aiming at understanding whether the Yap8-specific amino acids of the basic region contribute to Yap8-DNA binding, we mutated all these residues to Ala. The ability of the Yap8 mutant proteins to bind DNA was then assessed using the assay described above (Figure 2A). Only the mutation of Leu26 to Ala impaired growth in the absence of His (Figure 4A). Further corroborating this result, we showed that this mutation inhibits binding to the Y8RE, as evaluated by EMSA using total protein extracts (Figure 4C). These results also explain the growth behavior of the *yap8* strain overexpressing *YAP*8L26A, *YAP*8S29A and *YAP*8N31A in the presence of arsenic compounds. Indeed, solely *YAP8*L26A gene was not able to rescue the arsenic sensitive phenotype of the *yap8* mutant (Figure 4B). Further corroborating this result, the mutation of Leu26 to Ala severely compromises the expression of *ACR2* gene, one of the Yap8 targets, paralleling the mutation of the conserved residue Arg22 (Figure 4D). The loss of Yap8 activity in the *yap8* L26A mutant was not due to a destabilization of the protein, as the levels of the mutant and native protein are similar in the presence or absence or arsenic (Figure S2). 

Overall these results suggest that at least Leu26 is required for Yap8 DNA binding and that Asn31 is not required for Y8RE recognition.

### Yap8-specific residues Leu26 and Asn31 are required for Yap8 specificity

Although Yap8 exhibits a high similarity with other Yap family members at the level of the bZIP domain, Yap8 is the least related to the other Yap members. Indeed, Yap8 only recognizes the Y8RE [11] in contrast to Yap1 that recognizes multiple sites [4,6,7]. Aiming at understanding whether the Yap8-specific amino acids, Leu26, Ser29 and Asn31, are responsible for its specificity these residues were mutated to the ones present in Yap1- Asn, Ala and Arg, respectively (Figure 1A). We then tested the ability of the mutants to bind the Y8RE and YRE *in vivo*, using the approach depicted in Figure 2A.

**Figure 5:**
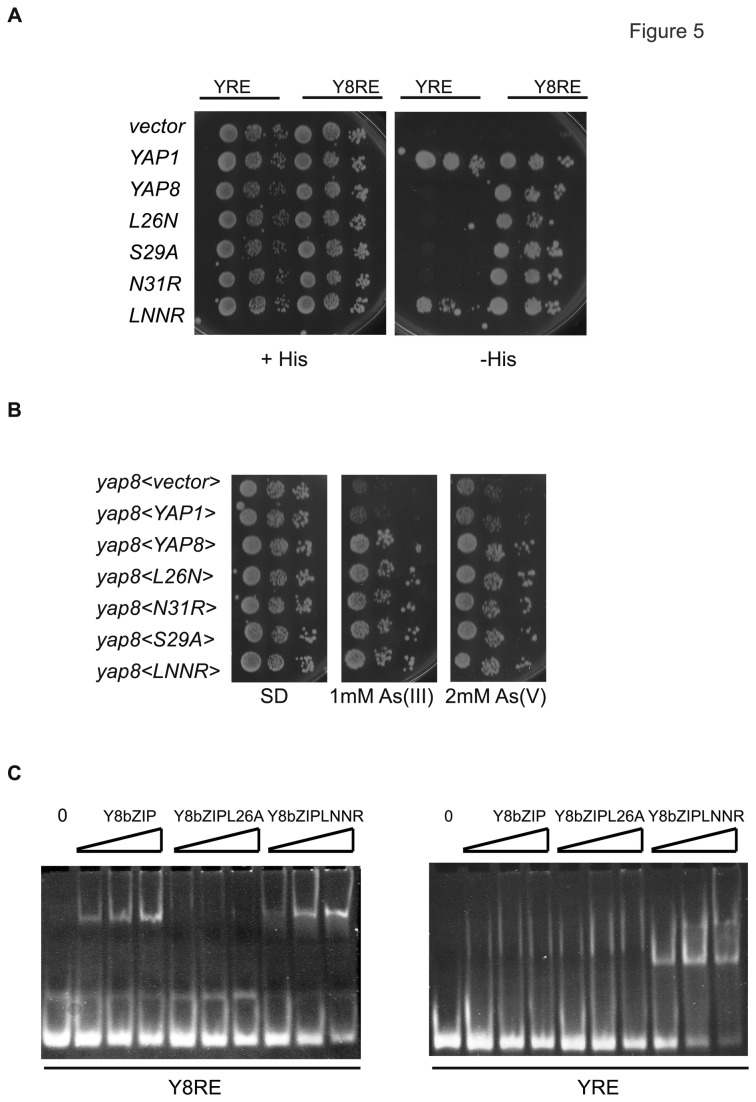
Yap8-specific amino acids Leu26 and Asn31 narrow Yap8 specificity. (**A**) Single replacement of Yap8-specific amino acids by Yap1 residues does not impair Yap8 response element (Y8RE) recognition, whereas double substitution allows Yap response element (YRE) recognition by Yap8. Strains overexpressing the indicated Yap or Yap8 mutated versions and containing the HIS3 under the control of the YRE (TATTACTAATC) or the Y8RE (TGATTAATAATCA) were spotted onto selective media with or without histidine (+His or -His). (**B**) Yap8 activity is not affected by replacement of Yap8-specific amino acids for the corresponding residues of Yap1. Wild-type (A) and *yap8* mutant strains (B) were transformed respectively with the empty plasmid YEplac112 (<vector>), the plasmids overexpressing the native YAP8, and the plasmid containing the YAP8 mutated versions. Serially diluted cells were spotted onto selective dropout, as indicated. (**C**) The Yap8bZIPLNNR mutated protein is capable of binding the Y8RE *in* *vitro*. Purified Yap8bZIP and Yap8 mutated proteins (Yap8bZIPL26A and Yap8bZIPLNNR) were incubated with the oligomers containing either the Yap response element (YRE) or the Y8RE and analyzed by electrophoretic mobility shift assay (EMSA), as described in materials and methods.

As previously shown, replacement of Ser29 by Ala did not disturb the Yap8 mutant binding to DNA (Figure 4A and Figure 5A). Moreover, substitution of Asn31 by Arg had no effect in the binding to Y8RE (Figure 5A). A similar result was observed following mutation of this residue to Ala (Figure 4A). Contrary to the results observed when Leu26 was mutated to Ala (Figure 4A), replacement of Leu26 to Asn did not prevent Y8RE recognition (Figure 5A). Accordingly, the *yap8* mutant strain transformed with each of these mutated genes grew in the presence of arsenic (Figure 5B). 

None of the single mutations of *YAP*8 allowed the recognition of the YRE (Figure 5A). As such, we decided to simultaneously replace two of the Yap8-specific amino acids, Leu26 and Asn31, by the corresponding residues in Yap1. The resultant Yap8 double mutant of Yap8L26N-N31R (Yap8LNNR) was capable of growing in the absence of His (Figure 5A), suggesting that these two amino acids in Yap1 (Asn74 and Arg79) are relevant for Yap1 site recognition. In addition, this mutant protein also recognizes the Y8RE and is able to rescue the growth phenotype of a *yap8* strain (Figure 5A and Figure 5B). The levels of the Yap8LNNR mutant protein were similar to the wild-type Yap8 protein, in the presence or absence of arsenic (Figure S2). The protein level of Yap8-specific mutants does not vary, when compared to the wild-type Yap8 protein (Figure S2).

To further confirm the relevance of Leu26 and Asn31 residues in Y8RE and YRE recognition we mutated the above residues in the Yap8bZIP creating the double mutant Yap8L26N-N31R to assess the purified protein binding to the Y8RE and YRE, through EMSA. We used the wild-type protein, Yap8bZIP and Yap8bZIPL26A mutant as a positive and negative control respectively. Corroborating the *in vivo* assays (Figure 5A), EMSA revealed that the wild-type Yap8bZIP interacts only with the Y8RE, whereas the double mutant Yap8bZIPLNNR is capable of recognizing both Yap8 and Yap1 response elements (Figure 5C). As expected the Yap8bZIPL26A mutant, does not interact with either the YRE or the Y8RE (Figure 5C). 

We next raised the hypothesis that the double mutant Yap8bZIPLNNR could recognize the Y8RE with less affinity than the wild-type protein. To test this possibility, surface plasmon resonance (SPR) was used to estimate the kinetic constants (*k*_*a*_, association rate constant; *k*_*d*_, dissociation rate constant; and *K*_D_, dissociation constant) of the formation of complexes established between Yap8bZIP, Yap8bZIPLNNR and Yap8bZIPL26A and the Y8RE. The sensograms obtained were used to estimate the kinetic constants. Remarkably, Yap8bZIP and the double mutant Yap8bZIPLNNR have similar affinities to the Y8RE (similar *K*_D_, Table 1). Although the dissociation constants are the same, the kinetics of binding is different. The values obtained for the Yap8bZIPLNNR mutant show that it associates to DNA and dissociates slower than Yap8bZIP (lower *k*_*a*_ and *k*_*d*_ values for the Yap8bZIPLNNR mutant when compared to Yap8bZIP) (Table 1). As expected, and corroborating the above described results (Figure 4), Yap8bZIPL26A mutant has less affinity for the Y8RE sequence (*K*_D_ 17.2 nM) when compared to Yap8bZIP (K_*D*_ 6.1 nM). This complex associates at a similar rate of the Yap8bZIPLNNR-DNA complex (similar *k*_*a*_), although it dissociates more rapidly (*k*_*d*_ 5.8x10^-3^ *versus k*_*d*_ 1.5x10^-3^, Table 1).

**Table 1:** Binding affinity of the different Yap8bZIP proteins for the Y8RE.

Protein	*K*_D_ (nM)	*k*_a_ (1/Ms) (x10^5^)	*k*_d_ (1/Ms) (x10^-3^)
**Yap8bZIP**	6.1 ± 1.8	11.3 ± 1.7	9.0 ± 1.2
**Yap8bZIPL26A**	17.2 ± 1.9	3.3 ± 0.4	5.8 ± 1.3
**Yap8bZIPLNNR**	6.5 ± 0.4	2.2 ± 0.4	1.5 ± 0.3

The affinity of Yap8bZIP proteins to the Y8RE was compared by surface plasmon resonance. The association and dissociation rates (*k*_a_ and *k*_d_ respectively) of complexes established between the 25-bp DNA sequence of the *ACR3* promoter harboring the Y8RE and the Yap8bZIP proteins were obtained by fitting the sensograms to a Langmuir 1:1 binding model as detailed in the materials and methods.

Altogether the results suggest that Yap8 specific amino acids contribute to Yap8 specificity, possibly because Leu26 together with Asn31 hamper Yap1-site recognition by Yap8. Furthermore our results point to a role of these two amino acids in the stability of the Yap8-DNA complex.

## Discussion

The yeast bZIP transcription factor Yap8 plays a pivotal role in the response to arsenic stress, being the major regulator of the arsenate reductase (*ACR2*) and arsenite efflux pump gene (*ACR3*) [8,9,11,25]. Yap8 and Yap1 have a similar basic region predicted to bind DNA [4]. Nevertheless, Yap1 barely activates transcription of *ACR2* and *ACR3*, whereas Yap8 does not recognize the YRE. In the present work, we have attempted to understand the mechanisms responsible for the specificity of Yap8. 

Using homology modeling, a model of the complex Yap8bZIP-DNA was built with the Pap1-DNA crystal structure acting as a template [18]. The model showed several conserved and Yap8-specific residues that may be close to the DNA (Figure 1D). To test the model predictions these residues were mutated to Ala and the ability of the mutants to bind the Y8RE and to respond to arsenic stress were assessed using *in vivo* and *in vitro* genetic and biochemical approaches. Regarding the conserved residues of the Yap8 basic region, our experimental data corroborated the bioinformatics data. Indeed, all the conserved residues predicted to interact with the DNA (Arg22, Gln25, Arg27, Arg36) were shown to be important for Yap8 DNA binding (Figure 2B and 2C). Indeed, these four mutations that, *in vivo*, do not bind Y8RE also do not reveal significant changes in the transactivation potential, when compared to the wild-type (Figure S3). The EMSA assay indicates that although Yap8bZIPQ25A, Yap8bZIPR27A and Yap8bZIP36A are able to bind the Y8RE, the signal of the complex is less intense than the one observed in the wild-type Yap8bZIP protein (Figure 2C). 

Overexpression of the Yap8 Ala mutants of Arg27 and Arg36 into a *yap8* mutant strain was still able to rescue the growth phenotype in the presence of arsenic (Figure 2D). The apparent disparity between Figure 2B and 2D may be explained by the fact that the threshold for DNA binding needed to activate the *ACR2* and *ACR3* is lower than the one required for the activation of the *HIS3* reporter gene. We therefore, expected that the contribution of Arg27 and Arg36 to Yap8-DNA interaction would not be as relevant as those of Arg22 and Gln25. Indeed, the Yap8bZIP-DNA model suggests that although Gln25, Arg27 and Lys36 side chains are close to the backbone phosphates, Gln25 is also in the proximity of Arg22 that is involved in direct contacts with a base (Figure 1D) similar to what is observed in Pap1. As such, it might be that the growth phenotype exhibited by the mutant Yap8Q25A is due to the perturbation of Arg22 orientation and not solely related with the loss of interaction with DNA phosphates.

Concerning the Yap8-specific amino acid residues Leu26, Ser29 and Asn31, only the substitution of Leu26 by Ala disrupted both Yap8 binding to the DNA and Yap8 activity (Figure 4). It is possible that the side chain of the Leu has a hydrophobic contact with the methyl group of the thymine, that base pairs with the second adenine (TGATTAATAATCA, Figure 3). The small size of Ala is not sufficient for such an interaction. There are several other examples of hydrophobic contacts between protein and DNA. In Gcn4 bZIP transcription factor, Ala238 and Ala239 are in contact with the methyl group of thymines [21,23]. Also, in P22 c2 repressor of the bacteriophage P22, a Val residue (Val33) is found in a hydrophobic pocket composed of the methyl groups of four thymines [26]. 

Our experimental data clearly indicate that the specific residue Asn31 is not relevant for Y8RE recognition. However, we cannot unambiguously evaluate the relevance of Ser29 since its mutation to Ala effectively recreates part of the Yap1 basic region that when overexpressed was shown to bind the Y8RE (Figure 5A).

To address the increased specificity of Yap8 in respect to Yap1, we replaced the Yap8-specific amino acids by the corresponding residues in Yap1 and assayed the ability of the mutants to recognize the Y8RE and YRE. All the single mutants were able to recognize the Y8RE, but none of them bound the YRE (Figure 5A). Surprisingly, substitution of Leu26 by Asn did not disrupt binding to the Y8RE (Figure 5A), as occurred upon replacement by Ala (Figure 4A). This observation may be due to the fact that Asn and Leu occupy similar space and therefore Asn can have a similar packing with thymine. Interestingly, the double replacement of Leu26 and Asn31 for Asn and Arg, respectively, renders Yap8 capable of recognizing the Y8RE as well as the YRE (Figure 5A). This result indicates that, these two Yap8-specific amino acids are responsible for the restriction of Yap8 specificity. 

Moreover, we found that Yap1 is able to recognize the Y8RE (Figure 5A). This finding, together with the fact that Yap1 nuclear sequestration is activated under arsenic stress, raises the question of why this transcription factor does not regulate *ACR2* and *ACR3* expression in *yap8* mutant strains (Figure 5B). It has been demonstrated that *TRX2* induction by Yap1 only occurs after Yap1 correct activation by hydrogen peroxide that triggers the formation of two intra-molecular disulfide bonds between its N-and C-terminal cysteine-rich domains [27,28]. Although a mutant form of Yap1, with a mutation in the C-terminal domain, is nuclear resident, it does not recruit the mediator subunit Rox3, and thus does not induce *TRX2* expression [27]. One possibility is that arsenic compounds may interact with the cysteine rich domain of Yap1 and thus impede Yap1 from recruiting the required transcriptional machinery to the *ACR2* and *ACR3* promoters. On the other hand, using SPR assays we show that both Yap8bZIP and Yap8bZIP double mutant LNNR that is closer to the Yap1 basic region, bind the Y8RE with a similar affinity (Table 1). Notwithstanding, the stability of the complex Yap8bZIPLNNR-DNA is different from the one of Yap8bZIP-DNA (Table 1), since the double mutant associates and dissociates slower with the DNA. As such, another possibility is that Yap1 may have a lower association rate constant compared to Yap8, which *in vivo* would prevent Yap1 binding to *ACR2* and *ACR3* promoters.

## Supporting Information

Figure S1 **Protein levels of Yap8 conserved mutated versions.** *yap8* cells carrying the proteins Yap8 or the mutated Yap8 versions tagged with the epitope c-myc were grown in the absence or presence of 1mM of arsenate and Yap8 protein levels were evaluated by immunoblot, as described in the supporting materials and methods.
(TIF)

Figure S2 **Protein levels of Yap8-specific mutated versions.** *yap8* cells carrying the proteins Yap8 or the mutated Yap8 versions, tagged with the epitope c-myc, were grown in the absence or presence of 1mM of arsenate and Yap8 protein levels were evaluated by immunoblot, as described in the supporting materials and methods.
(TIF)

Figure S3 **Transactivation of the reporter gene *lacZ* by the Ala conserved amino acids mutants fused to lexA.** The plasmid harboring the construction *lexAYAP8* and mutated versions were transformed in a yeast strain, together with a plasmid containing eight lexA binding sites. After 1h treatment with 2mM As (V), the cells were collected and β-galactosidase activity was measured as described in supporting materials and methods. The graphic represents the mean of five or more, biological replicates and the corresponding standard deviation.
(TIF)

Figure S4 **Yap8bZIPK37A is capable of binding the Y8RE as the wild-type Yap8bZIP.** Total extracts of *E.coli* harboring the empty vector or the vector encoding the indicated protein (Y8bZIP or mutated versions) were obtained as described in materials and methods. After treatment with RNase, equal amounts of total protein were incubated with the same amount of the DNA Y8RE and were analyzed by Electrophoretic mobility shift assay (EMSA) as described in materials and methods.
(TIF)

Figure S5 **Far-UV circular dichroism spectra of the Yap8bZIP at 25°C and 4°C.** There is an increase in the α-helical content of the protein when the temperature is decreased from room temperature to 4°C. Protein in sodium phosphate buffer 10mM, pH 7.5 with 100mM of NaCl.
(TIF)

Text S1 **Supporting data including supporting methods and supporting tables.**
(DOC)

## References

[1] Rodrigues-Pousada CA, Nevitt T, Menezes R, Azevedo D, Pereira J et al. (2004) Yeast activator proteins and stress response: an overview. FEBS Lett 567: 80-85. doi:10.1016/j.febslet.2004.03.119. PubMed: 15165897. 15165897

[2] Azevedo D, Tacnet F, Delaunay A, Rodrigues-Pousada C, Toledano MB (2003) Two redox centers within Yap1 for H2O2 and thiol-reactive chemicals signaling. Free Radic Biol Med 35: 889-900. doi:10.1016/S0891-5849(03)00434-9. PubMed: 14556853. 14556853

[3] Harshman KD, Moye-Rowley WS, Parker CS (1988) Transcriptional activation by the SV40 AP-1 recognition element in yeast is mediated by a factor similar to AP-1 that is distinct from GCN4. Cell 53: 321-330. doi:10.1016/0092-8674(88)90393-5. PubMed: 2834068. 2834068

[4] Fernandes L, Rodrigues-Pousada C, Struhl K (1997) Yap, a novel family of eight bZIP proteins in Saccharomyces cerevisiae with distinct biological functions. Mol Cell Biol 17: 6982-6993. PubMed: 9372930. 9372930 10.1128/mcb.17.12.6982 PMC232555

[5] Goudot C, Etchebest C, Devaux F, Lelandais G (2011) The reconstruction of condition-specific transcriptional modules provides new insights in the evolution of yeast AP-1 proteins. PLOS ONE 6: e20924. doi:10.1371/journal.pone.0020924. PubMed: 21695268. 21695268 PMC3111461

[6] He XJ, Fassler JS (2005) Identification of novel Yap1p and Skn7p binding sites involved in the oxidative stress response of Saccharomyces cerevisiae. Mol Microbiol 58: 1454-1467. doi:10.1111/j.1365-2958.2005.04917.x. PubMed: 16313629. 16313629 PMC2916641

[7] Nguyên DT, Alarco AM, Raymond M (2001) Multiple Yap1p-binding sites mediate induction of the yeast major facilitator FLR1 gene in response to drugs, oxidants, and alkylating agents. J Biol Chem 276: 1138-1145. doi:10.1074/jbc.M008377200. PubMed: 11056165. 11056165

[8] Menezes RA, Amaral C, Delaunay A, Toledano M, Rodrigues-Pousada C (2004) Yap8p activation in Saccharomyces cerevisiae under arsenic conditions. FEBS Lett 566: 141-146. doi:10.1016/j.febslet.2004.04.019. PubMed: 15147884. 15147884

[9] Wysocki R, Fortier PK, Maciaszczyk E, Thorsen M, Leduc A et al. (2004) Transcriptional activation of metalloid tolerance genes in Saccharomyces cerevisiae requires the AP-1-like proteins Yap1p and Yap8p. Mol Biol Cell 15: 2049-2060. doi:10.1091/mbc.E03-04-0236. PubMed: 14978214. 14978214 PMC404003

[10] Wysocki R, Bobrowicz P, Ułaszewski S (1997) The Saccharomyces cerevisiae ACR3 gene encodes a putative membrane protein involved in arsenite transport. J Biol Chem 272: 30061-30066. doi:10.1074/jbc.272.48.30061. PubMed: 9374482. 9374482

[11] Ilina Y, Sloma E, Maciaszczyk-Dziubinska E, Novotny M, Thorsen M et al. (2008) Characterization of the DNA-binding motif of the arsenic-responsive transcription factor Yap8p. Biochem J 415: 467-475. doi:10.1042/BJ20080713. PubMed: 18593383. 18593383

[12] Ghosh M, Shen J, Rosen BP (1999) Pathways of As(III) detoxification in Saccharomyces cerevisiae. Proc Natl Acad Sci U S A 96: 5001-5006. doi:10.1073/pnas.96.9.5001. PubMed: 10220408. 10220408 PMC21806

[13] Menezes RA, Amaral C, Batista-Nascimento L, Santos C, Ferreira RB et al. (2008) Contribution of Yap1 towards Saccharomyces cerevisiae adaptation to arsenic-mediated oxidative stress. Biochem J 414: 301-311. doi:10.1042/BJ20071537. PubMed: 18439143. 18439143

[14] Wemmie JA, Szczypka MS, Thiele DJ, Moye-Rowley WS (1994) Cadmium tolerance mediated by the yeast AP-1 protein requires the presence of an ATP-binding cassette transporter-encoding gene, YCF1. J Biol Chem 269: 32592-32597. PubMed: 7798263. 7798263

[15] Bouganim N, David J, Wysocki R, Ramotar D (2001) Yap1 overproduction restores arsenite resistance to the ABC transporter deficient mutant ycf1 by activating ACR3 expression. Biochem Cell Biol 79: 441-448. doi:10.1139/bcb-79-4-441. PubMed: 11527213. 11527213

[16] Tzamarias D, Struhl K (1994) Functional dissection of the yeast Cyc8-Tup1 transcriptional co-repressor complex. Nature 369: 758-761. doi:10.1038/369758a0. PubMed: 8008070. 8008070

[17] Sali A (1995) Comparative protein modeling by satisfaction of spatial restraints. Mol Med Today 1: 270-277. doi:10.1016/S1357-4310(95)91170-7. PubMed: 9415161. 9415161

[18] Fujii Y, Shimizu T, Toda T, Yanagida M, Hakoshima T (2000) Structural basis for the diversity of DNA recognition by bZIP transcription factors. Nat Struct Biol 7: 889-893. doi:10.1038/82822. PubMed: 11017199. 11017199

[19] Laskowski RA, MacArthur MW, Moss DS, Thornton JM (1993) PROCHECK: a program to check the stereochemicai quality of protein structures. J Appl Crystallography 26: 283-291. doi:10.1107/S0021889892009944.

[20] Lu XJ, Olson WK (2003) 3DNA: a software package for the analysis, rebuilding and visualization of three-dimensional nucleic acid structures. Nucleic Acids Res 31: 5108-5121. doi:10.1093/nar/gkg680. PubMed: 12930962. 12930962 PMC212791

[21] Ellenberger TE, Brandl CJ, Struhl K, Harrison SC (1992) The GCN4 basic region leucine zipper binds DNA as a dimer of uninterrupted alpha helices: crystal structure of the protein-DNA complex. Cell 71: 1223-1237. doi:10.1016/S0092-8674(05)80070-4. PubMed: 1473154. 1473154

[22] Di Y, Tamás MJ (2007) Regulation of the arsenic-responsive transcription factor Yap8p involves the ubiquitin-proteasome pathway. J Cell Sci 120: 256-264. doi:10.1242/jcs.03346. PubMed: 17200139. 17200139

[23] Keller W, König P, Richmond TJ (1995) Crystal structure of a bZIP/DNA complex at 2.2 A: determinants of DNA specific recognition. J Mol Biol 254: 657-667. doi:10.1006/jmbi.1995.0645. PubMed: 7500340. 7500340

[24] Weiss MA, Ellenberger T, Wobbe CR, Lee JP, Harrison SC et al. (1990) Folding transition in the DNA-binding domain of GCN4 on specific binding to DNA. Nature 347: 575-578. doi:10.1038/347575a0. PubMed: 2145515. 2145515

[25] Bobrowicz P, Wysocki R, Owsianik G, Goffeau A, Ułaszewski S (1997) Isolation of three contiguous genes, ACR1, ACR2 and ACR3, involved in resistance to arsenic compounds in the yeast Saccharomyces cerevisiae. Yeast 13: 819-828. doi:10.1002/(SICI)1097-0061(199707)13:9. PubMed: 9234670. 9234670

[26] Watkins D, Hsiao C, Woods KK, Koudelka GB, Williams LD (2008) P22 c2 repressor-operator complex: mechanisms of direct and indirect readout. Biochemistry 47: 2325-2338. doi:10.1021/bi701826f. PubMed: 18237194. 18237194

[27] Gulshan K, Rovinsky SA, Coleman ST, Moye-Rowley WS (2005) Oxidant-specific folding of Yap1p regulates both transcriptional activation and nuclear localization. J Biol Chem 280: 40524-40533. doi:10.1074/jbc.M504716200. PubMed: 16219769. 16219769

[28] Delaunay A, Isnard AD, Toledano MB (2000) H2O2 sensing through oxidation of the Yap1 transcription factor. EMBO J 19: 5157-5166. doi:10.1093/emboj/19.19.5157. PubMed: 11013218. 11013218 PMC302088

