# Supplementary figures and images for: Two Residues in the Basic Region of the Yeast Transcription Factor Yap8 Are Crucial for Its DNA-Binding Specificity
Source: PLoS One. 2013 Dec 16;8(12):e83328. doi: 10.1371/journal.pone.0083328 (PMC3865217; doi:10.1371/journal.pone.0083328)

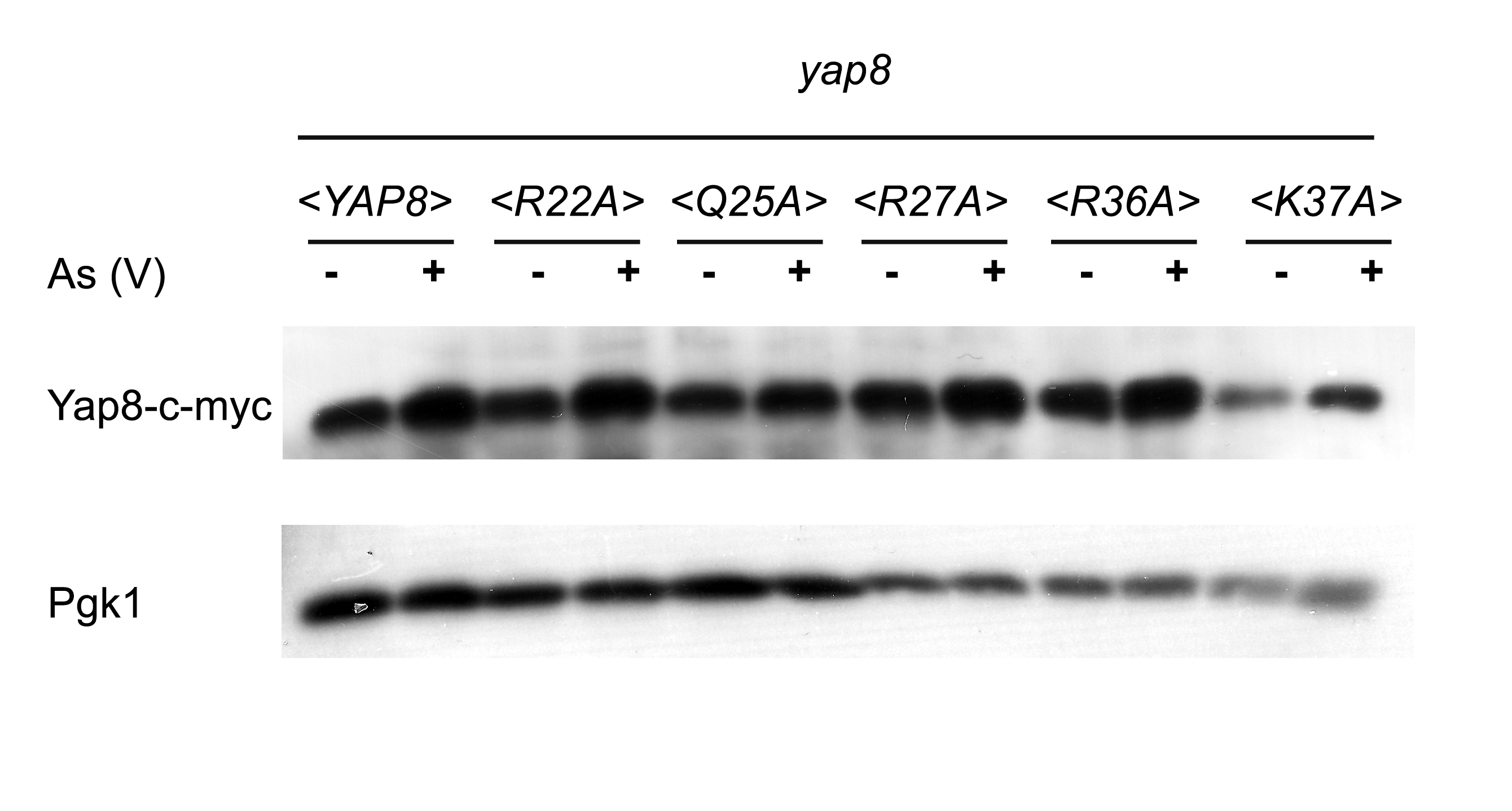

Supplement: Figure S1 — Protein levels of Yap8 conserved mutated versions. yap8 cells carrying the proteins Yap8 or the mutated Yap8 versions tagged with the epitope c-myc were grown in the absence or presence of 1mM of arsenate and Yap8 protein levels were evaluated by immunoblot, as described in the supporting materials and methods. (TIF) [file pone.0083328.s001.tif]

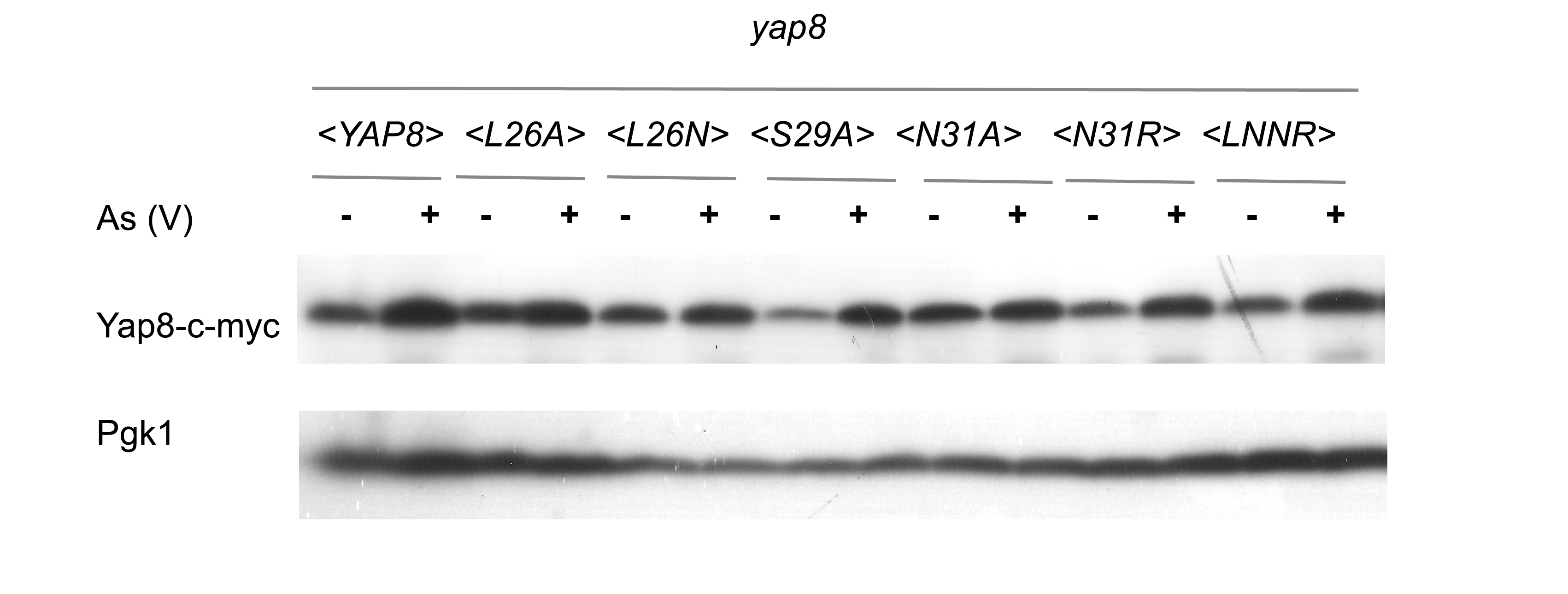

Supplement: Figure S2 — Protein levels of Yap8-specific mutated versions. yap8 cells carrying the proteins Yap8 or the mutated Yap8 versions, tagged with the epitope c-myc, were grown in the absence or presence of 1mM of arsenate and Yap8 protein levels were evaluated by immunoblot, as described in the supporting materials and methods. (TIF) [file pone.0083328.s002.tif]

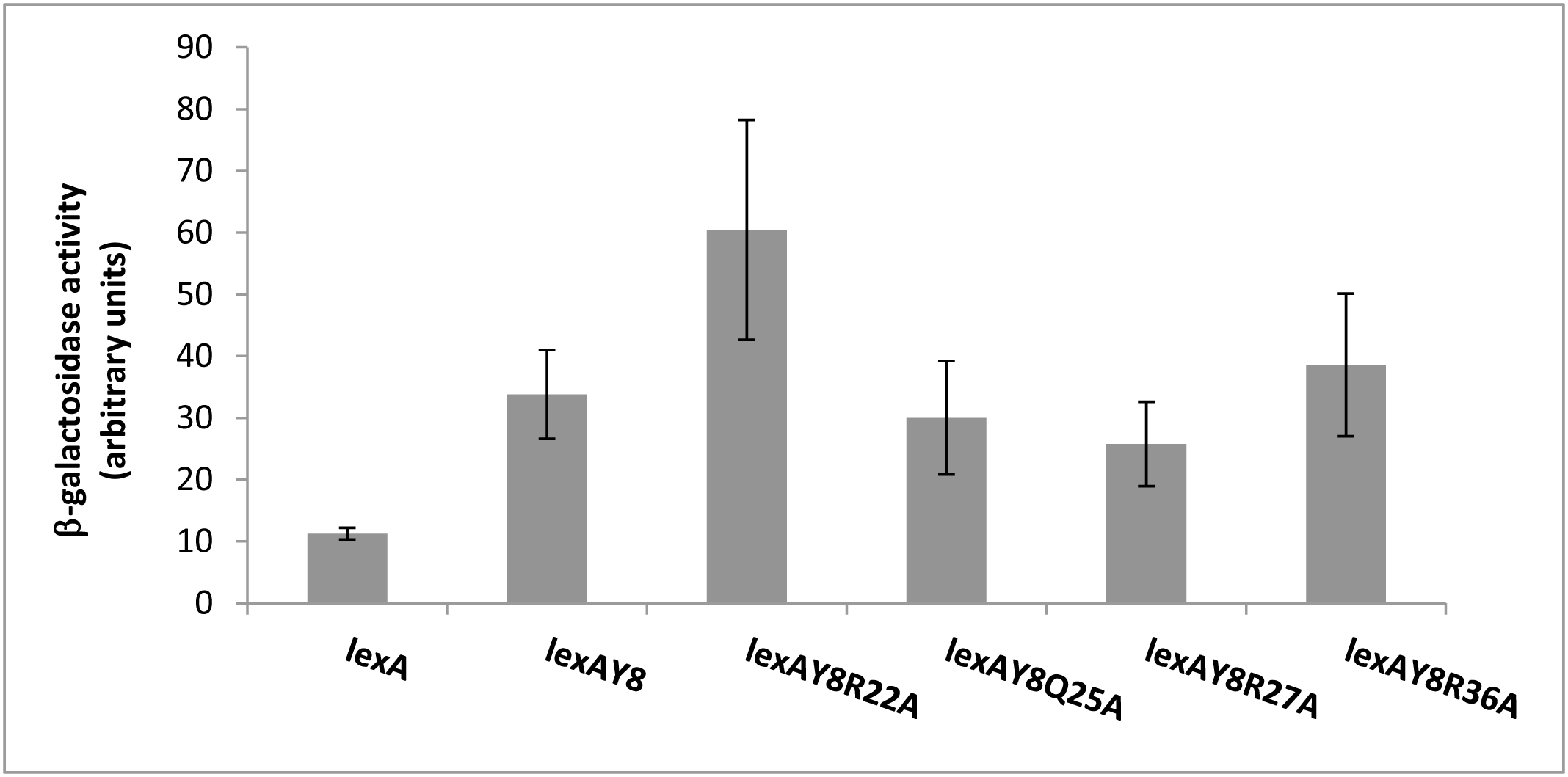

Supplement: Figure S3 — Transactivation of the reporter gene lacZ by the Ala conserved amino acids mutants fused to lexA. The plasmid harboring the construction lexAYAP8 and mutated versions were transformed in a yeast strain, together with a plasmid containing eight lexA binding sites. After 1h treatment with 2mM As (V), the cells were collected and β-galactosidase activity was measured as described in supporting materials and methods. The graphic represents the mean of five or more, biological replicates and the corresponding standard deviation. (TIF) [file pone.0083328.s003.tif]

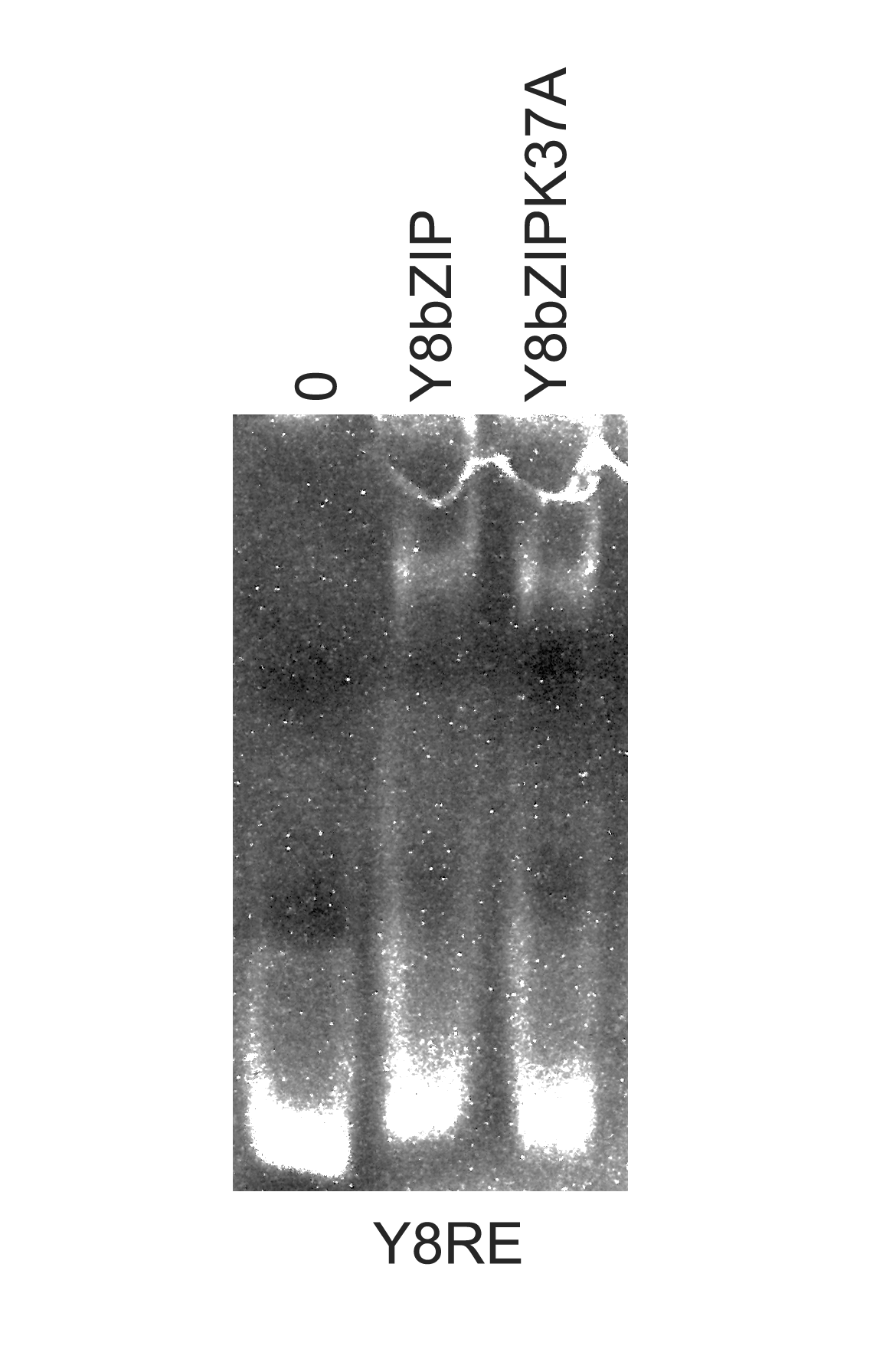

Supplement: Figure S4 — Yap8bZIPK37A is capable of binding the Y8RE as the wild-type Yap8bZIP. Total extracts of E.coli harboring the empty vector or the vector encoding the indicated protein (Y8bZIP or mutated versions) were obtained as described in materials and methods. After treatment with RNase, equal amounts of total protein were incubated with the same amount of the DNA Y8RE and were analyzed by Electrophoretic mobility shift assay (EMSA) as described in materials and methods. (TIF) [file pone.0083328.s004.tif]
