# Supplementary material for: Two Residues in the Basic Region of the Yeast Transcription Factor Yap8 Are Crucial for Its DNA-Binding Specificity
Source: PLoS One. 2013 Dec 16;8(12):e83328. doi: 10.1371/journal.pone.0083328 (PMC3865217; doi:10.1371/journal.pone.0083328)
Supplement: Text S1 — Supporting data including supporting methods and supporting tables. (DOC) [file pone.0083328.s006.doc]

**Supporting information**

**Supporting Materials and Methods**

**Immunoblot Assays**

FT4 *yap8* strain was transformed with the plasmids pRS416 Y8-c-myc, or the indicated mutated versions and the resulting cells were grown to early exponential phase, induced with 1mM Na_2_HAsO_4_ .7H_2_0 (As(V)) (Sigma) and harvested after 1h. Protein extracts were prepared using trichloroacetic acid and 50µg of total protein were run in a 10% polyacrylamide gel and immunoblotted as described previously [1]. The 9E10 anti-c-Myc monoclonal antibody (Roche) was used to detect Yap8. Pgk1 (Invitrogen) was used as a loading control [2]. The signal was detected using SuperSignal (Thermo Scientific).

**CD spectroscopy**

Circular dichroism (CD) spectra were measured with a JASCO J-815 spectropolarimeter with Peltier temperature control. An average of 10 accumulations were recorded in the far-UV region (190 to 260nm) at 25ºC and 4ºC with a data pitch of 0.2nm and a polarized quartz cuvette with 0.1-cm-path-length. A bandwidth of 2nm was used with a detector response of 1s and scanning speed of 200nm/min. The protein spectra were obtained in 10mM phosphate buffer (pH 7.5) with 100mM NaCl and afterwards corrected by subtracting the spectrum of the buffer solution.

**β-Galactosidase Activity Assay**

The FT4 *yap8* [3] deleted mutant strain was co-transformed with the 2µ plasmid pSH18-34, which carries the lacZ reporter gene controlled by eight lexA operators, and the plasmid encoding lexAYap8 fusion (wild-type or mutated versions). The cells were grown in SD liquid medium to exponential phase and were treated for 1h with 2mM Na_2_HAsO_4_ .7H_2_O (As(V)) (Sigma). The harvested cells were ressuspended in LacZbuffer( 60mM Na_2_HPO_4_, 40mM NaH_2_PO_4_, 10Mm KCl and 1mM MgSO_4_, pH7) and permeabilized with chloroform. Relative enzyme activity was assayed by measuring at A.420nm the degradation of the substrate ONPG (*o-nitrophenyl-β-D-galactoryranoside*) (Sigma) and normalized against cell content as measured at O.D.600nm.

**Supporting tables**

| **name** | **Description** | **source** |
| --- | --- | --- |
| YAP8 mutants overexpression | | |
| YEplac112 | 2µ, *TRP1* | [4] |
| Yap1 | Yap1 in YEplac112 | [5] |
| Yap8 | Yap8 in YEplac112 | This study |
| Y8R22A | Yap8 mutant in YEplac112 | This study |
| Y8Q25A | Yap8 mutant in YEplac112 | This study |
| Y8L26A | Yap8 mutant in YEplac112 | This study |
| Y8L26N | Yap8 mutant in YEplac112 | This study |
| Y8R27A | Yap8 mutant in YEplac112 | This study |
| Y8N31A | Yap8 mutant in YEplac112 | This study |
| Y8N31R | Yap8 mutant in YEplac112 | This study |
| Y8S29A | Yap8 mutant in YEplac112 | This study |
| R36Y8A | Yap8 mutant in YEplac112 | This study |
| R37Y8A | Yap8 mutant in YEplac112 | This study |
| Y8LNNR | Yap8 mutant in YEplac112 | This study |
| DNA binding assay | | |
| pYap site | *his3*-Δ101,189 with Yap site inserted between *Eco*RI and *Bam*HI site as the only UAS of the *HIS3* | [5] |
| pYap8 site | *his3*-Δ101,189Yap8 site inserted between *Eco*RI and *Bam*HI site as the only UAS of the *HIS3* | This study |
| Yap8 in centromeric plasmid for immunoblotting and qRT-PCR | | |
| pRS416 | *CEN*, *URA3* | Stratagene |
| pRS416 Y8-c-myc |  | This study |
| pRS416 Y8L26A-c-myc | pRS416 Y8**-**c**-**myc with L26A mutation | This study |
| pRS416 Y8LNNR-c-myc | pRS416 Y8**-**c**-**myc with L26N-N31R mutation | This study |
| Yap8 bZIP for EMSA and SPR | | |
| Yap8bZIP | Yap8 bZIP (from bp 30 to 195) in pET30a(+) | This study |
| Yap8LA26AbZIP | Yap8 bZIP with L26A mutation | This study |
| Yap8LNNRbZIP | Yap8 bZIP with L26N-N31R mutation | This study |
| lexAYap8 for trans-activation potential measurements | |  |
| lexAYap8 | Yap8 in YCp91 [5] | [6] |
| lexAYap8R22A | Yap8 mutant in YCp91 [5] | This study |
| lexAYap8Q25A | Yap8 mutant in YCp91 [5] | This study |
| lexAYap8R27A | Yap8 mutant in YCp91 [5] | This study |
| lexAYap8R36A | Yap8 mutant in YCp91 [5] | This study |

**Table S1.** Plasmids used in the present study.

| **Oligomers** | **sequence 5’ to 3’** |
| --- | --- |
| YAP8 mutagenesis |  |
| Y8R22A.fw | AAA AAT AAG GCA GCT GCG CAA |
| Y8R22A.rv | TTG CGC AGC TGC CTT ATT TTT |
| Y8Q25A.fw | AGA GCT GCG GCA CTT AGA GCA |
| Y8Q25A.rv | TGC TCT AAG TGC CGC AGC TCT |
| Y8L26A.fw | AGC TGC GCA AGC TAG AGC ATC C |
| Y8L26A.rv | GGA TGC TCT AGC TTG CGC AGC T |
| Y8L26N.fw | AGC TGC GCA AAA TAG AGC ATC C |
| Y8L26N.rv | GGA TGC TCT ATT TTG CGC AGC T |
| Y8R27A.fw | GCA ACT TGC AGC ATC CCA AA |
| Y8R27A.rv | TTT GGG ATG CTG CAA GTT GC |
| Y8N31A.fw | GCA TCC CAA GCA GCA TTT AGa |
| Y8N31A.rv | TCT AAA TGC TGC TTG GGA TGC |
| Y8N31R.fw | AG CAT CCC AAA GAG CAT TTA GA |
| Y8N31R.rv | TC TAA ATG CTC TTT GGG ATG CT |
| Y8S29A.fw | CTT AGA GCA GCC CAA AAC GCA |
| Y8S29A.rv | TGC GTT TTG GGC TGC TCT AAG |
| R36Y8A.fw | CAT TTA GAA AAG CAA AGT TGG |
| R36Y8A.rv | TTC CAA CTT TGC TTT TCT AAA |
| Y8K37A.fw | AGA AAA CGA GCG TTG GAA AGA |
| Y8K37A.rv | TCT TTC CAA CGC TCG TTT TCT |
| Construction of Yap8-c-myc | |
| Yap8-1000XbaI.fw | GACTTCTAGACATCAAGTCAAGCCATGACA |
| ClaIT | TCC ATC GA TGA CAC AGC GCA ACA TTG TC |
| Y8TERM.rv | CAT CTA GA GTCG ACC CTA GTA CAA GAC CGC C |
| A1N2 | CTA GGC AAC TCA AGG GCC |
| Construction of Yap8bZIP |  |
| Yap8bZipNdeI fw | GGAATTC CATATG AGG AAG CCT TCA CTT AC |
| Yap8bZipXhoI rv | CGGctcgagACCCTGAAAATATAAATTTTCCTTCTTTAATATGTGAATTT |
| Electrophoretic Mobility Shift Assay | |
| Y8RE Fw | gatcc TGATTAATAATCAg |
| Y8RE Rv | aattc TGATTATTAATCA g |
| YRE Fw | gatcc GAATAAGTAAGCG g |
| YRE Rv | aattc CGCTTACTAATTC g |
| Surface Plasmon Resonance | |
| pACR3_25_Fw | TTTGTTTGATTAATAATCAACTTTA |
| pACR3_25_Rv | TAAAGTTGATTATTAATCAAACAAA |
| q-RT-PCR |  |
| qRTACR2-Fw | AGGCAACTCAAGGCCTAAT |
| qRTACR2-Rv | GAACATGCCAAGCGTTTGTA |
| ACT1_Fw | CTA TTG GTA ACG AAA GAT TCA |
| ACT1_Rv | CCT TAC GGA CAT CGA CAT CA |

**Table S2.** Oligomers used in the present study.

| **protein** | **alignment** |
| --- | --- |
| **Pap1**  **Yap8** | DQEPSSKRKAQNRAAQRAFRKRKEDHLKALETQVVTLKELHSSTTLENDQLRQKVRQLEEELRILEPS-----  --PPKNKRAAQLRASQNAFRKRKLERLEELEKKEAQLTVTND------------------------------- |
| **Pap1**  **Yap8** | ----S-KRKAQNRAAQRAFRKRKEDHLKALETQVVTLKELHSSTTLENDQLRQKVRQLEEELRIL --------QPPKNKRAAQLRASQNAFRKRKLERLEELEKKEAQLTVTNDQ------------------------------ |

**Table S3.** Alignment of Yap8 protein basic region sequence with the protein sequence of the Pap1 crystallized region, used for the model generation.

**Supporting References:**

1. Nevitt T, Pereira J, Rodrigues-Pousada C (2004) YAP4 gene expression is induced in response to several forms of stress in Saccharomyces cerevisiae. Yeast 21: 1365-1374.

2. Kou H, Zhou Y, Gorospe RM, Wang Z (2008) Mms19 protein functions in nucleotide excision repair by sustaining an adequate cellular concentration of the TFIIH component Rad3. Proc Natl Acad Sci U S A 105: 15714-15719.

3. Menezes RA, Amaral C, Batista-Nascimento L, Santos C, Ferreira RB, et al. (2008) Contribution of Yap1 towards Saccharomyces cerevisiae adaptation to arsenic-mediated oxidative stress. Biochem J 414: 301-311.

4. Gietz RD, Sugino A (1988) New yeast-Escherichia coli shuttle vectors constructed with in vitro mutagenized yeast genes lacking six-base pair restriction sites. Gene 74: 527-534.

5. Fernandes L, Rodrigues-Pousada C, Struhl K (1997) Yap, a novel family of eight bZIP proteins in Saccharomyces cerevisiae with distinct biological functions. Mol Cell Biol 17: 6982-6993.

6. Menezes RA, Amaral C, Delaunay A, Toledano M, Rodrigues-Pousada C (2004) Yap8p activation in Saccharomyces cerevisiae under arsenic conditions. FEBS Lett 566: 141-146.
